# Hugl1 and Hugl2 in Mammary Epithelial Cells: Polarity, Proliferation, and Differentiation

**DOI:** 10.1371/journal.pone.0047734

**Published:** 2012-10-23

**Authors:** Atlantis Russ, Jeanne M. V. Louderbough, Daniela Zarnescu, Joyce A. Schroeder

**Affiliations:** 1 Genetics Interdisciplinary Program, University of Arizona, Tucson, Arizona, United States of America; 2 Department of Molecular and Cellular Biology, University of Arizona, Tucson, Arizona, United States of America; 3 Arizona Cancer Center, University of Arizona, Tucson, Arizona, United States of America; 4 BIO5 Institute, University of Arizona, Tucson, Arizona, United States of America; Sanford Burnham Medical Research Institute, United States of America

## Abstract

Loss of epithelial polarity is described as a hallmark of epithelial cancer. To determine the role of Hugl1 and Hugl2 expression in the breast, we investigated their localization in human mammary duct tissue and the effects of expression modulation in normal and cancer cell lines on polarity, proliferation and differentiation. Expression of Hugl1 and Hugl2 was silenced in both MCF10A cells and Human Mammary Epithelial Cells and cell lines were grown in 2-D on plastic and in 3-D in Matrigel to form acini. Cells in monolayer were compared for proliferative and phenotypic changes while acini were examined for differences in size, ability to form a hollow lumen, nuclear size and shape, and localization of key domain-specific proteins as a measure of polarity. We detected overlapping but distinct localization of Hugl1 and Hugl2 in the human mammary gland, with Hugl1 expressed in both luminal and myoepithelium and Hugl2 largely restricted to myoepithelium. On a plastic surface, loss of Hugl1 or Hugl2 in normal epithelium induced a mesenchymal phenotype, and these cells formed large cellular masses when grown in Matrigel. In addition, loss of Hugl1 or Hugl2 expression in MCF10A cells resulted in increased proliferation on Matrigel, while gain of Hugl1 expression in tumor cells suppressed proliferation. Loss of polarity was also observed with knockdown of either Hugl1 or Hugl2, with cells growing in Matrigel appearing as a multilayered epithelium, with randomly oriented Golgi and multiple enlarged nuclei. Furthermore, Hugl1 knock down resulted in a loss of membrane identity and the development of cellular asymmetries in Human Mammary Epithelial Cells. Overall, these data demonstrate an essential role for both Hugl1 and Hugl2 in the maintenance of breast epithelial polarity and differentiated cell morphology, as well as growth control.

## Introduction

Changes in cell polarity are required to establish a multitude of cellular fates, including differentiation, proliferation, migration, adhesion, and transformation of normal epithelium [1]. An extensive body of genetic and molecular research has identified three major protein complexes that function in a common pathway to regulate the establishment and maintenance of apicobasal polarity in epithelial tissues: Crumbs, Par and Scribble (Scrib, Lgl, Dlg) complexes [Reviewed in [2], [3]]. Responding to internal and external signals, these three complexes engage in an elegant interplay to create polar domains within the plasma membrane, separating it into apical and basolateral territories guarded by tight junctions. The Crumbs and Par complexes localize to the apical surface, promoting apical membrane identity, while the Scribble complex localizes to the basolateral surface, promoting basolateral membrane identity. The complexes interact with one another through multiple protein-protein interaction sites and phosphorylation events that result in mutual exclusion of complexes from opposite domains [4], [5], [6], [7]. In an individual cell, membrane domains created by interactions between these protein complexes provide a framework for the positioning of other functional proteins throughout the membrane [2]. This is important for partitioning of growth factors and growth factor receptors [5], [8], [9] and it is also critical for the positioning of cell fate determinants in asymmetric stem cell division [10].

Components of the Scribble complex are considered neoplastic tumor suppressors, as their mutations in *Drosophila* epithelial and neural tissues cause loss of apicobasal polarity, overproliferation, and a failure to differentiate with many characteristics of metastatic growth including upregulation of proteases and increased invasive capability [6]. Functional conservation from *Drosophila* to humans is evidenced by the ability of human *HUGL1* to rescue the *Drosophila* loss of function phenotype when expressed exogenously [11]. Note that Lgl is designated as Hugl1 and Hugl2 in humans, Llgl in mice and Lgl in flies.

Biochemical experiments have identified Lgl as a cytoskeletal protein that localizes at the cortex and cytoplasm, containing multiple WD-40 motifs that are involved in protein-protein interactions and multiple serine residues that serve as sites of phosphorylation [12]. aPKC phosphorylates Lgl at these serine residues resulting in release from the cortex at the confluence of the two domains [13], [14], [15], [16]. In addition, an increase in the efficiency of aPKC to phosphorylate Llgl2 induces a loss of polarity in MDCK cells [17]. Lgl has been shown to directly interact with nonmuscle myosin IIa and the t-SNARE, syntaxin 4, and has been implicated in protein trafficking to the basolateral membrane [18], [19], [20], [21]. Lgl has also been found to play a role in planar organization of the embryonic epidermis of *Drosophila*, an event dependent upon Disheveled and myosin [22].

Loss of Lgl in non-mammalian model organisms has been correlated with a variety of phenotypes, including the disruption of apicobasal polarity and hyperproliferation, among others [10], [23], [24], [25], [26]. Of note, in *Drosophila*, these phenotypes appear to be tissue-restricted, and the promotion of polarity and suppression of cell cycle are differentially regulated in different tissues [27]. In mouse models, knockout of *Llgl1* produces a neonatally fatal phenotype, with mice succumbing to hydrocephaly and neuroectodermal tumor formation in the brain [28]. In contrast, knockout of *Llgl2* is not lethal, yet results in defective branching morphogenesis in the placenta [29]. According to this study, no role for *Llgl2* as a tumor suppressor was observed, although the authors did not report evaluation of the mammary gland. Finally, in MDCK cells, both knockdown of Llgl2 and Llgl1/Llgl2 were reported to affect apical protein localization and polarization of cells in acinar culture [15]. Overall, these studies point to a general role for Lgl1 and Lgl2 in promoting apicobasal polarity in epithelial tissues.

In mammary gland epithelium, loss of polarity occurs during the progression to neoplasia. In a small study of primary breast tumors, 13 of 17 showed loss of Hugl1 expression by RT-PCR [11]. Hugl1 has been implicated as a tumor suppressor in the progression of colorectal cancer, endometrial cancer and malignant melanoma [30]. In all three cancers, studies showed an inverse correlation between Hugl1 expression and tumor progression. Reintroduction of Hugl1 into malignant melanoma cell lines resulted in increased adhesion, decreased invasive activity, downregulated matrix metalloproteinases (MMPs) and upregulated E-cadherin, supporting a role for Hugl1 in suppression of Epithelial to Mesenchymal Transition (EMT) [31]. Hugl2 expression is also lost in colorectal cancer and is downregulated in breast cancer, via *ZEB1*, a transcription factor that induces EMT [32]. These data indicate a possible role for the loss of either Hugl1 or Hugl2 in the progression of mammary gland epithelium from well-ordered and polarized ductal structures to precancerous lesions.

Given their conserved roles in growth suppression and polarity maintenance, we hypothesized that Hugl proteins were important in promoting mammary epithelial apicobasal polarity and a differentiated phenotype. We have examined the role of Hugl in the maintenance of polarity in ductal epithelial cells of the human breast and found that Hugl1 and Hugl2 control apicobasal polarity, cell morphology, and proliferation. Overall, these data demonstrate an essential role for Hugl1 and Hugl2 in maintenance of breast epithelial polarity and cell morphology and suggest that it may function as a gatekeeper between a proliferative and differentiated state.

## Methods

### Human Tissue Immunofluorescence

The study design was evaluated and approved by the Human Subjects Research and Institutional Review Board at the University of Arizona and it was determined that the study did not constitute human research, therefore the need for written informed consent from participants was waived. Deidentified normal human mammary tissues were obtained from the Tissue Acquisition and Cellular/Molecular Analysis Shared Service (TACMASS) of the Arizona Cancer Center. Tissues were dissected and fixed in 10% buffered formalin, embedded in paraffin and sectioned. Tissue was deparafinized with xylenes, washed in ethanol and rehydrated. Antigen retrieval was performed in boiling 1 mM EDTA. Tissues were incubated with anti-Hugl1, anti-Hugl2 and anti-cytokeratin 18 (H80) antibodies at a dilution of 1∶100 and 1∶200 respectively. Secondary antibodies were anti-mouse-594 (Invitrogen) and anti-rabbit-488 (Invitrogen) and were used at 1∶400 and 1∶200, respectively.

### Cell Culture

MCF10A cell lines were obtained from American Type Culture Collection (ATCC) and cultured in Dulbecco’s modified Eagle medium/F12 (DMEM/F12) supplemented with 5% Horse Serum (Invitrogen), 10 µg/ml insulin, 100 ng/ml Cholera toxin (Sigma Aldrich), 20 ng/ml Epidermal Growth Factor, 1% Pen-Strep (Cellgro), and 0.5 µg/ml Hydrocortisone. Primary HMEC cells were obtained from Invitrogen and cultured in serum-free HuMEC Ready Medium with HuMEC supplement, Bovine Pituitary Extract (Invitrogen), and 1% Pen-Strep. MDA-MB-453 cells were obtained from ATCC and cultured in RPMI media supplemented with 10% fetal bovine serum (Omega Scientific) and 1% Pen-Strep. All cells were grown at 37°C in 5% CO_2_.

### Transfections and Viral Transductions

MDA-MB-453 cells were seeded in 6 well plates at 3×10^5^ cells per well. Cell lines were transfected with pEGFP-Lgl1-C1 [33] and the empty vector p-EGFP-C1 (Clontech) using Lipofectamine 2000 transfection reagent (Invitrogen) as per the manufacturer’s specifications. Stable transfectants were selected with 1.5 mg/ml G418.

MISSION shRNA lentiviral particles containing nontarget control shRNA or Hugl1or Hugl2 shRNAs and packaging vectors were purchased from Sigma Aldrich (NM_004140, clones TRCN0000117137-141) (NM_004524, clones TRCN0000116432-436). For transduction, virus was added to MCF10A and HMEC cells at a multiplicity of infection (MOI) range of 1 to 5 in the presence of 8 µg/ml hexadimethrine bromide (Sigma Aldrich) in culture medium. Transduced cells were selected using puromycin dihydrochloride (Sigma Aldrich) at 2 µg/ml and 0.5 µg/ml for MCF10As and HMECs, respectively. Stable lines were used as heterogenous populations; clones were not selected.

### MTT Assays

Cells were plated in 96 well plates at a density of 5×10^3^ cells per well and grown for 72 hours. 100 µl of (3-(4,5-dimethylthiazol-2-yl)-2,5-diphenyltetrazolium bromide (MTT) in RPMI (1∶10 dilution) was added to each well and incubated at 37°C for 4 hours. After incubation, MTT solution was aspirated and 50 µl of Dimethyl sulfoxide (DMSO) was added to each well. The plates were read after 10 minutes using a µQuant plate reader (Biotek Instruments).

### Acinar Culture and Slide Preparation

Three dimensional culture assays of MCF10As on Growth Factor Reduced Matrigel (BD Biosciences) were performed according to the protocol previously described in [34]. In 8 chamber slides, a cell suspension of 6000 cells per well was overlayed onto a thin bed of Matrigel (45 µl per well) in assay medium. Cells were cultured for 8 or 21 days with Assay Medium, consisting of DMEM/F12 (Invitrogen) supplemented with 2% Horse Serum (Omega Scientific), 0.5 µg Hydrocortisone, 100 ng/ml Cholera Toxin, 10 µg/ml insulin, 1% Pen/Strep, 5 ng/ml EGF, and 2 µg/ml puromycin that was changed every four days.

HMECs were seeded into 8 chamber slides with Matrigel, as above, in a cell suspension of 8×10^3^ cells per well according to Invitrogen protocols. Cells were cultured for 6 or 10 days in HuMEC Serum Free Medium supplemented with HuMEC supplement, Bovine Pituitary Extract (Invitrogen), 1% Pen/Strep, 10% matrigel, and 0.5 µg/ml puromycin that was changed every two days.

### Light, Fluorescent, and Confocal Microscopy

Live acini in Matrigel and cells grown on plastic were visualized with a Leica DM IL microscope and images were captured with a mounted Nikon E4500 digital camera using a 10X objective at room temperature.

MCF10A acini grown for 8 days in matrigel were fixed, mounted with ProLong Gold antifade reagent with DAPI, and visualized with a 10X objective under UV excitation with a Zeiss AxioCam mounted to a Zeiss Axiovert 200 microscope. 10 images per treatment were taken from two wells each of an 8 chamber slide. Frames of each image were designated by dividing each well into five fields and taking one image per field. The length on longest axis of each acinus within a given field was measured in pixels with Image Pro Plus software.

For confocal imaging, cells cultured in Matrigel were fixed with 2% paraformaldehyde-PBS for 10 minutes, washed once with PBS, and permeabilized with a solution containing 0.5% Triton X-100, 10 mM Pipes (pH 6.8), 50 mM NaCl, 300 mM sucrose, and 3 mM MgCl_2_ for 5 minutes at room temperature. Following fixation, cells were blocked with 3% BSA containing 0.05% Tween (blocking buffer) for 30 minutes, and incubated at 4°C for 18 hours with primary antibody (1∶200) in blocking buffer. Cells were washed 10 times over 2 hours with 0.2% BSA, 0.05% Tween in PBS (washing buffer) and subsequently incubated at 4°C for 18 hours with secondary antibodies conjugated to fluorophores. Cells were washed 10 times over 2 hours and mounted with ProLong Gold anti-fade reagent containing DAPI (Invitrogen).

Acini and tissue slides were visualized and imaged at room temperature with a Leica SP5 confocal microscope using the 40X and 63X NA 1.4 oil immersion objective and LAS AF vs2.5.1.6757 software (Leica Microsystems). Confocal images of acini were taken of single equatorial sections by focusing on the largest diameter of each acinus within the depth of field. Image quality and channel intensity were adjusted with gain and offset correction using the Leica LAS AF software. Raw.lif images were exported as.tif files and were resized and adjusted for brightness and contrast in Photoshop software (Adobe). Individual image metafiles were archived.

### Western Blots

Cultured cells were lysed in ice-cold lysis buffer containing 20 mM TRIS pH 7.5, 150 mM NaCl, 1% NP40, 5 mM EDTA pH 8.0, 1% NaF, 1% NaVan, 0.1% NH4 Molybdate and 8% Complete phosphatase and protease inhibitor (Roche). The lysates were centrifuged at 13,000 rpm for 10 min at 4°C and supernatant was collected for Western blot analysis. 20 µg protein lysate was separated by SDS-PAGE (7%) and transferred to PVDF membrane (Millipore). The membrane was blocked in 5% milk in PBS/0.1% Tween solution and then used for immunoblotting. Proteins on the membrane were treated with Super Signal Chemiluminescent Substrate (Pierce), visualized on Imagetech-B film (American X-ray) and developed with a Konica SRX-101C.

### Antibodies

The primary antibodies anti-Llgl1 (911–1010, cat # H00003996-M01) and anti-Llgl2 (101–200, cat # H00003993-M06) antibodies were purchased from Abnova. Anti-GFP (FL, sc-8334), anti-αTubulin (TU-02, sc8035), and anti-cytokeratin 18 (H80) were purchased from Santa Cruz Biotechnologies. Laminin λ2 (D4B5) was purchased from Millipore, anti-GM130 (cat # 610823) was purchased from BD Transduction Laboratories, anti-Ki-67 antibody (clone MIB-1, M7240) was purchased from Dako, and anti-MUC1 (Ab5) and anti-cleaved caspase-3 (Asp175) were purchased from Cell Signaling. Fluorescein-phalloidin was purchased from Invitrogen/Molecular Probes and anti-Integrin α6 was a kind gift from Dr. Anne Cress at the University of Arizona. Secondary antibodies conjugated to horseradish peroxidase (HRP) goat-antimouse IgG-IgM HRP, goat anti-rabbit IgG HRP, and goat anti-mouse IgG HRP and secondary antibodies conjugated to fluorophores (Alexa Fluor 488 anti-mouse, Alexa Fluor 488 anti-rabbit, Alexa Fluor 594 anti-mouse and Alexa Fluor 647 anti-rabbit) were purchased from Invitrogen/Molecular Probes. Anti-hamster FITC, was purchased from Jackson ImmunoResearch Labs.

### Image Quantification

Confocal images were quantified using LAS AF Lite 2.6.0 freeware (Leica Microsystems). To measure luminal infilling nuclei were counted with the count tool and a ratio of peripheral nuclei to internal nuclei was calculated. Ki 67 stained acini were also quantified using the count tool, assigning a score to each acinus based on the number of nuclei that showed positive Ki67 staining. Nuclear size was measured using the length tool to determine the longest axis of the largest nucleus per acinus in microns. Laminin V localization was scored in individual confocal images as either polarized, with staining on the basolateral surface of the acini, or unpolarized, with staining on the inside of the acinus and/or luminally located. Cleaved caspase 3 stained images of single acini were scored as either cleaved caspase positive or cleaved caspase negative.

Statistical differences in acinar size, luminal filling, nuclear size, and Ki67 were calculated with a two-tailed Student’s t test for each of three trials per experiment. Categorical data on Laminin V polarization and cleaved caspase 3 activity were compared with 2×2 contingency tables and statistical significance was calculated with a Fisher’s exact test.

## Results

### Hugl 1 and Hugl2 have Restricted Expression in Breast Epithelium and Loss Induces a Mesenchymal Phenotype

**Figure 1:**
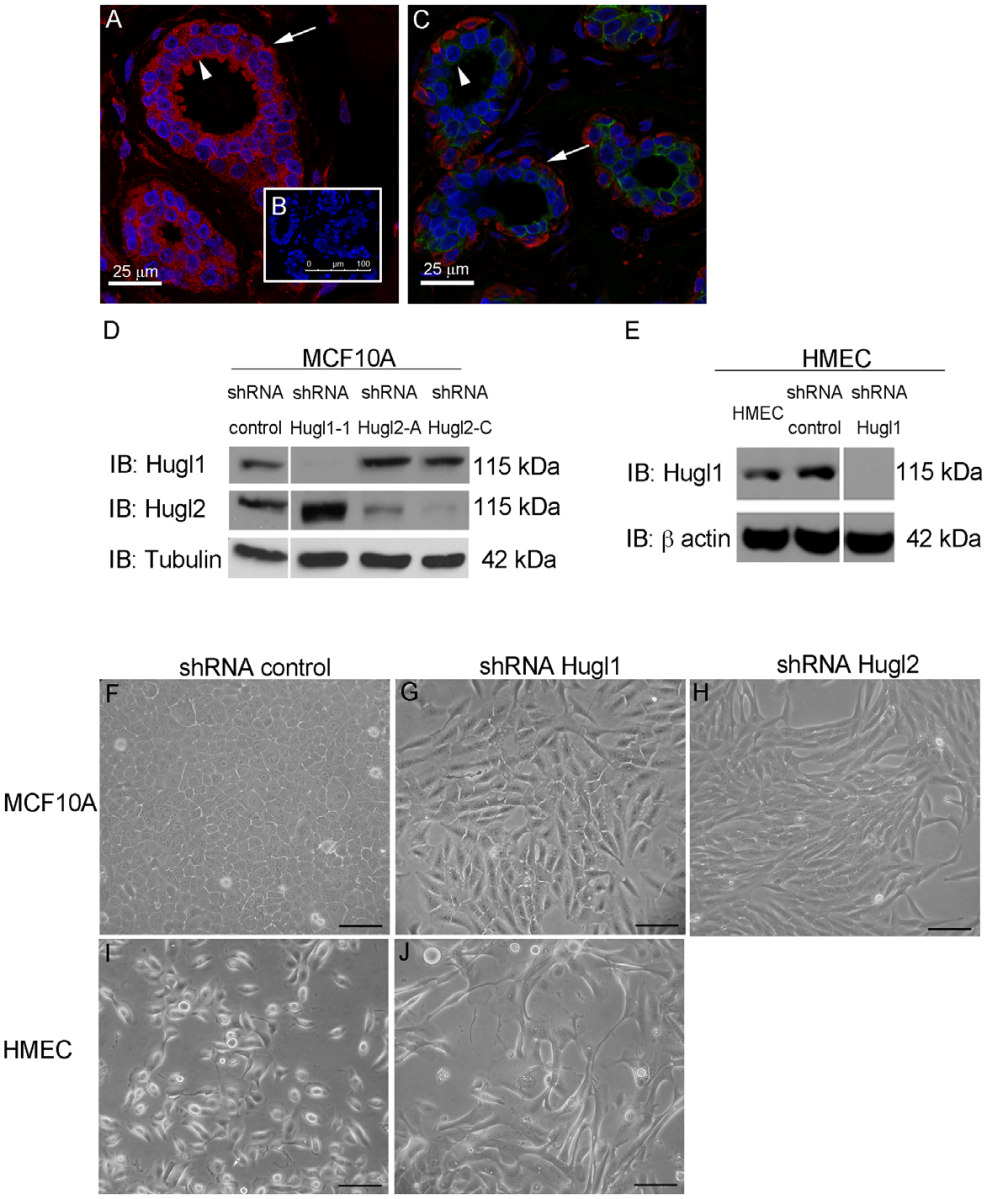
Hugl1 and Hugl2 display cell type restricted expression and inhibit mesenchymal phenotype. Ductal epithelium from human breast was incubated with antibodies to either (A) Hugl1 (red), or (C) Hugl2 (red) and Keratin 18 (green), or (B) no primary antibody, secondary only control. All sections were incubated with DAPI (blue). Arrowheads indicate luminal epithelium and arrows indicate myoepithelium. Stable knockdown was established in MCF10As and HMECs with transduction of (D and E) Hugl1, (H) Hugl2 or control shRNA lentiviral particles and selected with puromycin. Protein lysates were isolated from cell lines, 20 µg of protein were separated by SDS PAGE and analyzed by immunoblot using the antibodies: anti-Hugl1, anti-Hugl2, anti-β actin (loading control), and anti-α tubulin (loading control). Molecular weights are shown at right. MCF10A control shRNA cells (F) and HMEC control shRNA cells (I) retain parental cobblestone phenotype while Hugl1 shRNA cells (G and J) and shRNA Hugl2 cells (H) take on a mesenchymal phenotype on plastic after transduction. Scale bar = 300 µm.

Hugl is a core member of the Scribble complex, and in *Drosophila*, *lgl* can affect cell polarity and proliferation, although these events appear to be differentially regulated in different tissues [35]. Proliferative defects in *lgl* mutants in *Drosophila* have raised interest in the possibility for Hugl to confer tumor suppressive properties in humans. In humans, Hugl is represented by two genes, *HUGL1* and *HUGL2* which share 60% sequence homology, both of which are important for the function of the Scribble complex [15], [36]. To evaluate the role of Hugl1 and Hugl2 in breast tissue, we first evaluated normal breast tissue to determine whether each has distinct or overlapping expression in the luminal epithelium and myoepithelium. While Hugl1 was found in both the luminal and myoepithelium of the breast (Fig. 1, A), Hugl2 appeared to be largely restricted to the myoepithelial layer (Fig. 1, B, arrows indicate myoepithelium, Figure S1).

To evaluate the role of Hugl expression in mammary epithelium, Hugl1 and Hugl2 were each knocked down in either MCF10A cells (immortalized breast epithelium) or Human Mammary Epithelial Cells (HMECs, primary from breast reduction mammoplasty). To silence Hugl expression, five different short hairpin RNA (shRNA) sequences each were generated for Hugl1 and Hugl2 and knockdown was optimized in MCF10A cells and HMECs. Two shRNAs each for Hugl1 and Hugl2 resulted in optimal knockdown and similar phenotypes (Fig. 1, D and E, data for second Hugl1 shRNA, not shown). Effects of shRNA knockdown were specific to each paralog, as Hugl1 knockdown did not reduce Hugl2 expression or *vice versa* (Fig. 1, D). Subsequent to transduction, cells were selected with puromycin for one week before beginning experiments, and all experiments were performed on stably selected cell lines. In MCF10A cells, transduction of control shRNA yielded cells with a typical cobblestone morphology (Fig. 1, F). Notably, transduction of either Hugl1 or Hugl2 shRNA resulted in cells with mesenchymal phenotypes (elongated cells with extensive filopodia) (Fig. 1, G and H). Hugl1 knockdown in HMECs resulted in a similar mesenchymal phenotype (Fig. 1, J).

### Hugl1 and Hugl2 Control Acinar Formation in Matrigel

**Figure 2:**
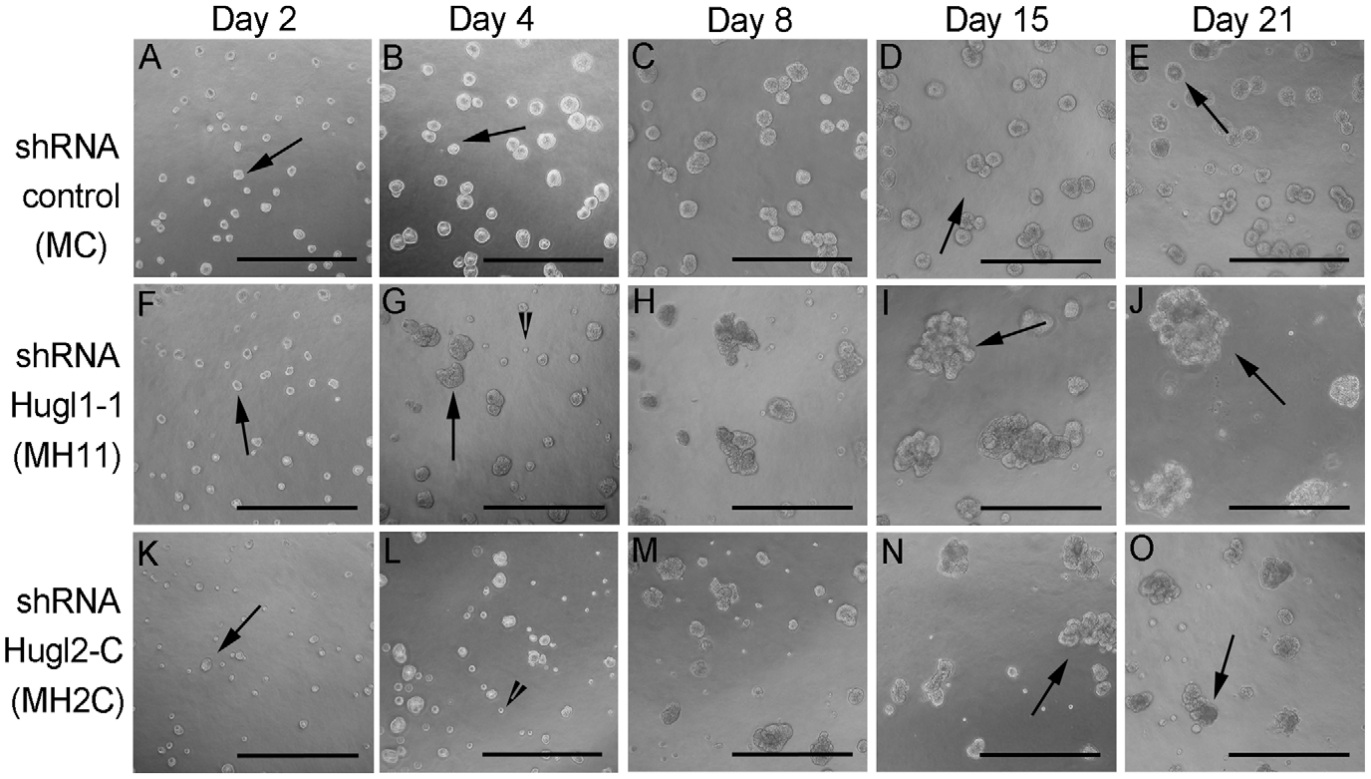
Hugl1 and Hugl2 promote regulated acini formation in mammary epithelia. Cells with stable expression of Hugl1-1 shRNA (MH11), Hugl2-C shRNA (MH2C) and control shRNA (MC) were cultured on Matrigel in 8 chamber slides for 21 days and imaged at 20X magnification by bright field. Arrows indicate acinar structures and arrowheads indicate abortive structures (A–O). Scale bar = 300 µm.

We next investigated whether Hugl loss in breast epithelial cells (immortalized MCF10A) would alter their ability to form polarized acini. To determine the effects of Hugl1 and Hugl2 protein loss on mammary epithelial polarity, MC (MCF10A-shRNA control), MH11 (MCF10A-shRNA Hugl1-1), or MH2C (MCF10A-shRNA Hugl2-C) cells were seeded in Matrigel (1.5×10^4^/ml) and grown for 21 days as previously described [34]. Immediately upon seeding (day 2), MH11 cells formed similar size and number colonies to MC cells while MH2C cells appeared unable to thrive (Fig. 2, A, F, K, arrows). By day 4, abortive structures could be observed in both MH11 and MH2Cs, but not in MCs (Fig. 2, B, G, L, arrowheads) [37]. These structures appeared to contain cells that survived in culture, yet did not expand to form acini. At day 15, large unorganized acinar overgrowths were obvious in both MH11 and MH2C, but not MC and these continued to grow through day 21 (Fig. 2, D, E, I, J, N and O, arrows).

**Figure 3:**
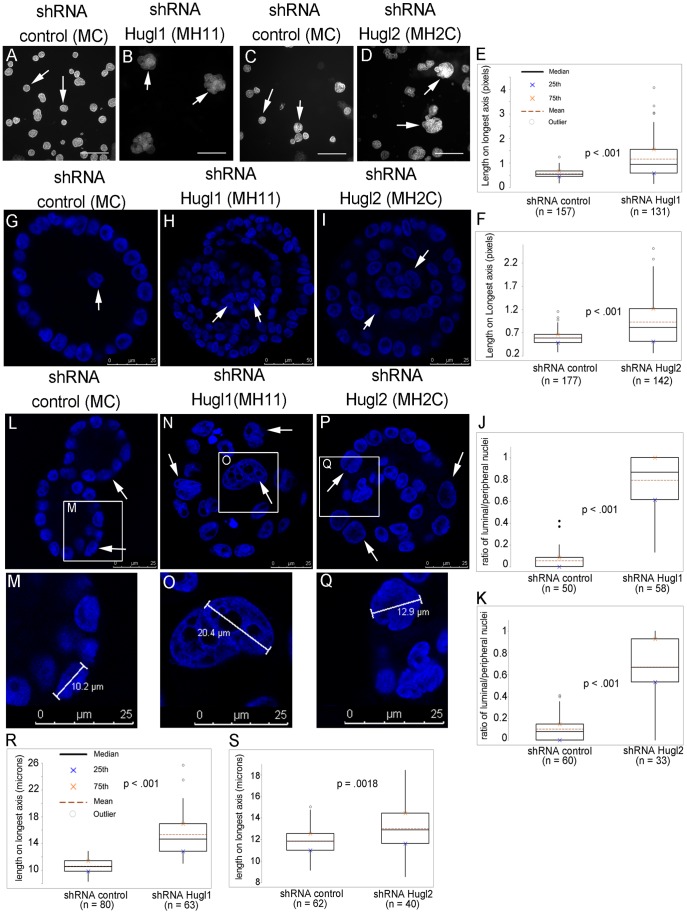
Hugl1 and Hugl2 regulate acinar size, nuclear size and lumen formation in MCF10As. Cells with stable expression of Hugl1-1 shRNA (MH11), Hugl2-C shRNA (MH2C) and control shRNA (MC) were grown in Matrigel for 21 days, fixed with 2% PFA, and incubated with 4′,6-diamidino-2-phenylindole (DAPI). (A–D) Images were taken at 10X (scale bar = 300 microns) and acini were measured for length on longest axis in pixels. Boxplots (E, F) reflect quantification of acinar size. For each experiment, boxplots reflect data from one of three trials producing similarly significant results. Arrows point to acini reflective of mean. (G–I, L–Q) Cross-sectional confocal images were taken of each acinus at 630X on a Leica confocal microscope. (G–I) A ratio of luminal nuclei/peripheral nuclei was calculated for each acinus to quantify infilling of the lumen. Arrows indicate luminal nuclei. (J–K) Boxplots reflect quantification of luminal infilling. (L–Q) Nuclear size was measured via length on longest axis in microns. White boxes outline magnified area (L, N, P). Nuclear measurements (M, O, Q) were recorded for each acinus and the largest value for each acinus was used to generate boxplots (R, S). Two-tailed Student’s t tests were performed to calculate p values. Scale bars indicate size in microns.

To evaluate the alterations to acinar size and structure that resulted from Hugl loss after 21 days of growth, slides were fixed in 2% paraformaldehyde (PFA) and incubated with 4′,6-diamidino-2-phenylindole (DAPI) (Fig. 3, A–D). The length of longest axis for every acinus in the field was measured in pixels (Fig. 3, A–D, arrows). For MH11, acinar size averaged 50% larger than MC (p<.001), while MH2C acinar size averaged 35% larger than MC (p<.001) (Fig. 3, E and F). Analysis of acini revealed distinct lumen formation in MC cells, but acinar infilling in both MH11 and MH2C. Confocal imaging of acini through the equatorial section (focused on largest cross sectional area), revealed largely empty acinar centers in MC, but highly cellular centers in both MH11 and MH2C with a multilayered-appearing acinar center (Fig. 3, G–I, arrows). To quantify cellular infilling, the ratio of luminal to peripheral cells was determined by counting nuclei. For MH11, luminal to peripheral nuclei averaged 70% greater than MC (p<.001) while MH2C luminal to peripheral nuclei averaged 53% greater than MC (p<.001) (Fig. 3, J and K). Analysis of acinar size revealed a striking difference in nuclear size as well. We observed increased nuclear size in MH11 and MH2C compared to MC, and this increase was seen in multiple cells within the acini (Fig. 3, L–Q, arrows). Nuclear size was quantified by measuring the length of the longest nuclear axis in microns (Fig. 3, M, O, Q). For MH11, nuclear size was 25% larger than MC (p<.001) while MH2C nuclear size was 11% larger than MC (p = .0018).

### Hugl1 and Hugl2 Inhibit Proliferation in Normal and Transformed Cells

**Figure 4:**
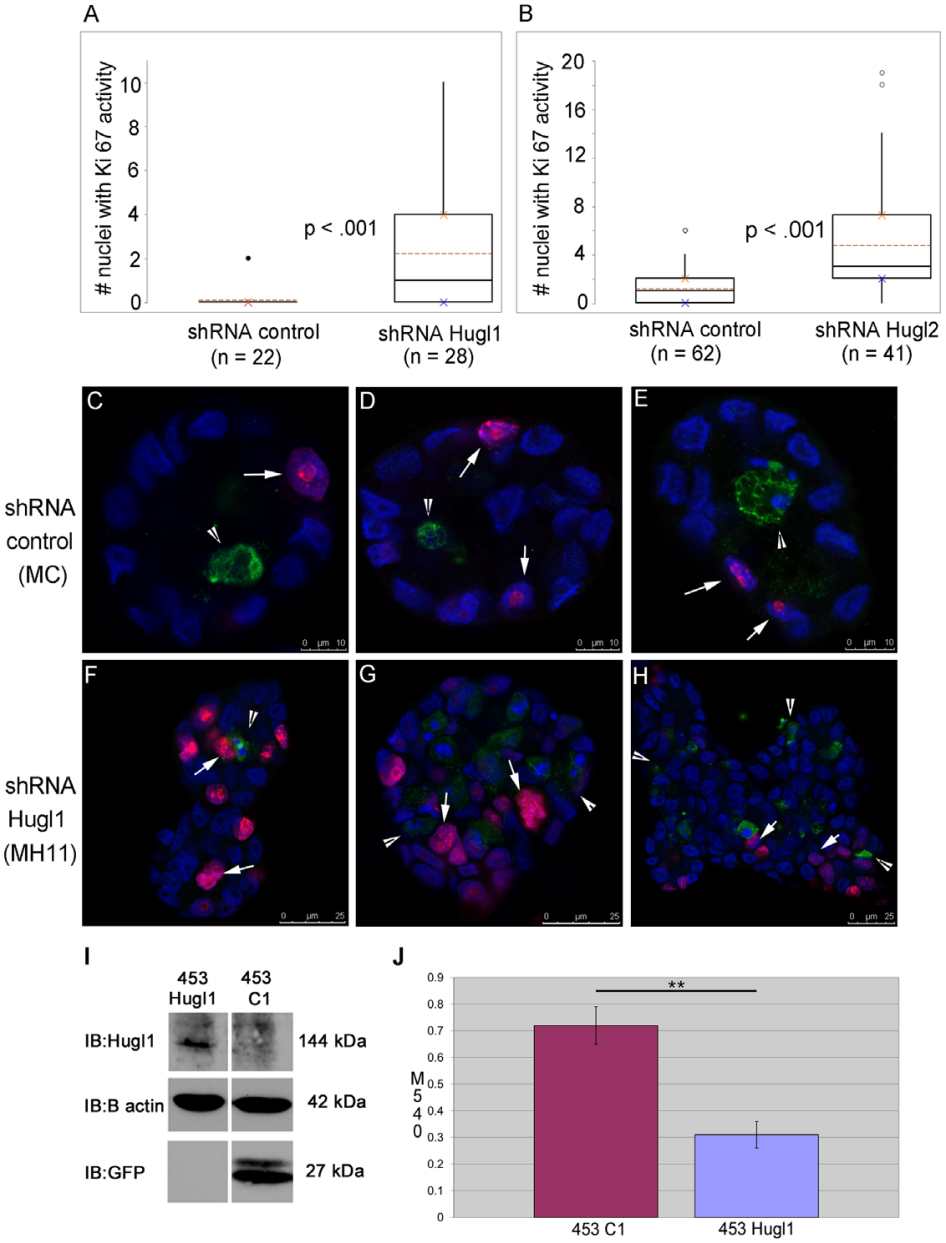
Hugl1 inhibits proliferation in both immortalized and transformed mammary epithelium. Cells with stable expression of Hugl1 shRNA (MH11), Hugl2 shRNA (MH2C) and control shRNA (MC) were grown in Matrigel for 8 days, fixed with 2% PFA, permeabilized, and incubated with anti-Ki 67 (proliferation) and/or anti- cleaved caspase 3 (apoptosis) antibodies. Cells were then incubated with secondary antibodies conjugated to fluorophores (Alexa 488, 647). Acini were imaged with a Leica confocal microscope to obtain cross-sectional images from the equatorial section at 630X. (A–B) Number of nuclei with Ki-67 activity was counted per acinus. For each experiment, boxplots reflect one of three trials producing similar statistically significant results. (C–H) Acini were immunofluorescently labeled for Ki-67 (fuschia, arrows) and cleaved caspase 3 activity (green, arrowheads). Scale bars indicate size in microns. (I) Re-expression of Hugl1 in breast cancer cell line MDA-MB-453 was established with stable transfection of a fusion protein construct, pEGFP-Hugl1 (453Hugl1). Control lines were transfected with empty vector pEGFP-C1 (453C1). 20 µg of protein lysate was separated by SDS PAGE and analyzed by immunoblot, probing for either anti-Hugl1 (top panel), anti-β actin (middle panel) or anti-GFP (bottom panel). Note that EGFP-Hugl1 is a fusion protein and is 144 KDa. Molecular weight is shown at right. (J) 453C1 and 453Hugl1 were grown for 3 days and analyzed by (3-(4,5-Dimethylthiazol-2-yl)-2,5-diphenyltetrazolium bromide (MTT) assay. Statistics were performed with two-tailed Student’s t test. ** p value <0.01.

As loss of Hugl dramatically affected acinar size in MCF10A cells, we next evaluated effects of Hugl expression on proliferation and/or apoptosis. MC, MH11, and MH2C were grown in Matrigel for 8 days, fixed, and analyzed by immunofluorescence for Ki-67 (marker of proliferation) and cleaved caspase 3 (marker of apoptosis). Quantification of Ki-67 positive nuclei revealed a 71% increase in MH11 versus MC (p<.001) and a 77% increase in MH2C versus MC (p<.001) (Fig. 4, A and B). Alternatively, analysis of the number of acini with active cleaved caspase 3 revealed a similar number of acini with caspase activity between MC and either MH11 or MH2C (Table S1).

Interestingly, the localization of Ki-67 and cleaved caspase 3 was also altered with loss of Hugl. In control cells, Ki-67 positive cells were restricted to the periphery (Fig. 4, C, D, E, arrow) and cleaved caspase 3 positive cells were restricted to the lumen in MC cells (Fig. 4, C, D, E, arrowhead); this was not the case with loss of Hugl1 or Hugl2. With MH11, Ki-67 and cleaved caspase 3 positive cells could be seen throughout the acini centers and peripheries (Fig. 4, F, G, H).

We next evaluated the effects of Hugl on cell proliferation in 2-D, as measured by 3-(4,5-Dimethylthiazol-2-yl)-2,5-diphenyltetrazolium bromide (MTT) assay. In contrast to what was observed on Matrigel, this analysis revealed no difference in growth between MC and MH11 (data not shown). To determine if Hugl1 expression could slow highly proliferative transformed cells, we took a gain of function approach and performed an MTT assay on MDA-MB-453 metastatic breast cancer cells, which lack endogenous Hugl1 expression. Cells were transfected with either a pEGFP-Hugl1 or a pEGFP-Control1 expression construct and selected with G418. Analysis of cells by GFP or Hugl1 antibodies demonstrated an induction of Hugl1 expression in 453-Hugl1 cells (Fig. 4, I). We found that 453-Hugl1 cells showed a 2-fold decrease in proliferation after 3 days compared to controls (Fig. 4, J). These data demonstrate that Hugl inhibits proliferation in MCF10A cells in an extracellular matrix-dependent manner, while effects on cancer cells are not dependent upon signals from the extracellular matrix.

### Hugl1 Controls Polarity in Mammary Epithelial Cells

**Figure 5:**
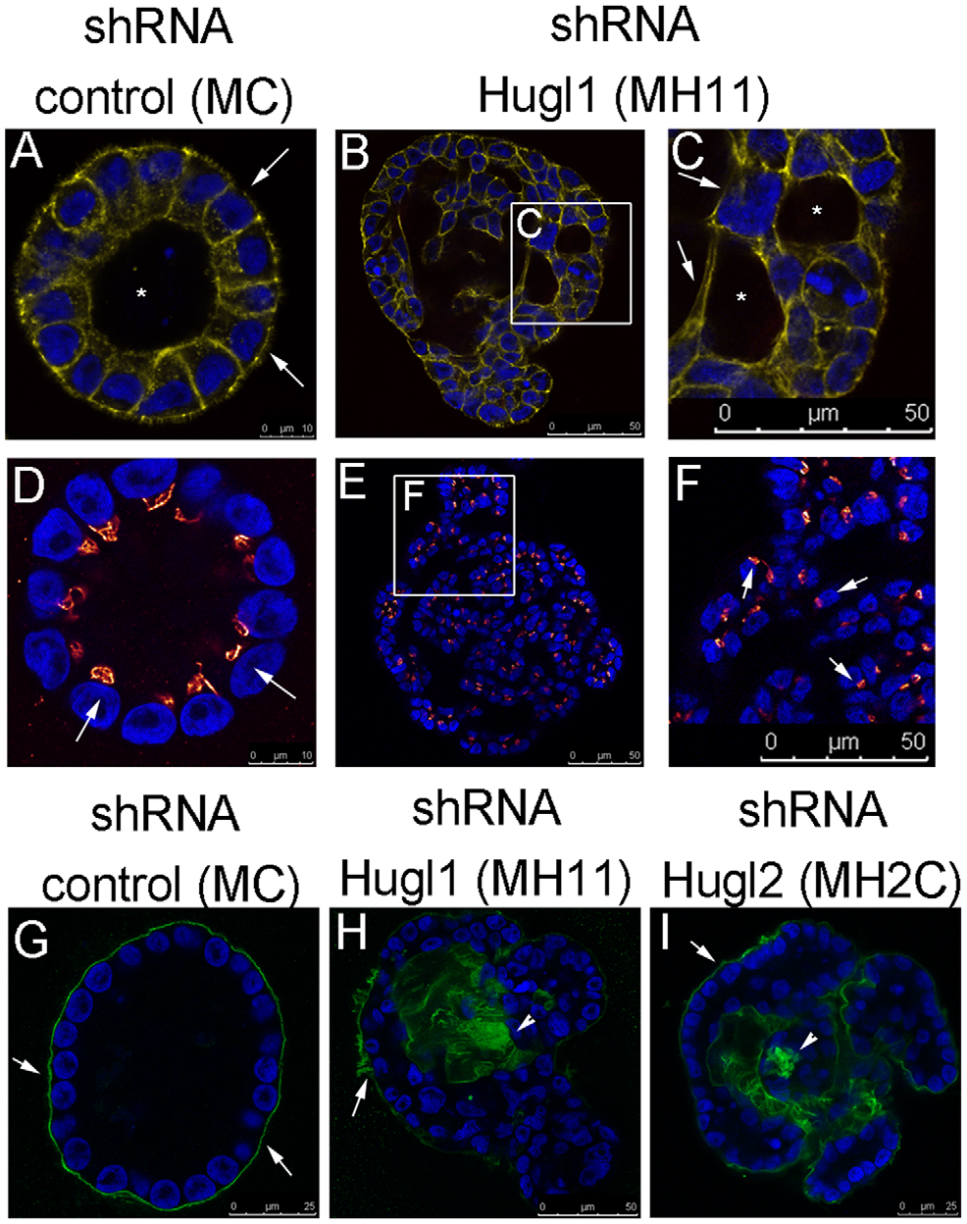
Hugl regulates apicobasal polarity and lumen formation in mammary acini. Cells with stable expression of Hugl1 shRNA (MH11), Hugl2 shRNA (MH2C) and control shRNA (MC) were cultured in Matrigel for 21 days, fixed with 2% PFA, permeabilized, and incubated with primary antibodies marking apical (anti-GM130, red), cytoskeletal (anti-phalloidin, yellow), or basal (anti-Laminin V, green) domains followed by fluorescently labeled secondary antibodies (FITC, Alexa 488, or Alexa 647). Slides were mounted with antifade mounting media containing DAPI. Acini were imaged with a Leica confocal microscope to obtain equatorial cross sectional images of their morphology. (A–I) All images were taken at 630X using internal zoom. Subsections of B and E (white boxes) magnified in C and F. Scale bars indicate size in microns.

To determine how loss of Hugl affects epithelial polarity during acini formation, markers of polarity were evaluated, including GM 130 (apical marker), Laminin V deposition (basal marker), and overall cellular structure (phalloidin to localize F actin). F-actin localization revealed uniform cytoskeletal structure of individual cells and a hollow lumen in MC acini (Fig. 5, A). Alternatively, in MH11 acini, cytoskeletal structure was irregular and multiple lumens could be observed (Fig. 5, B and C). Next, cells were incubated with an antibody to GM 130, which revealed apical presentation of the Golgi facing the single lumen in MC acini (Fig. 5, D, arrows). Alternatively, in MH11 acini, which lacked a single defined lumen, Golgi were oriented in a disorganized fashion (Fig. 5, E and F, arrows), indicating disruption of planar polarity. Finally, basement membrane deposition (Laminin V) in MC acini was exclusively basally localized, forming a ring on the outer edge of each acinus (Fig. 5, G, arrows). Alternatively, basement membrane deposition in both MH11 and MH2C was haphazard and could be found both basally (Fig. 5, H and I, arrows) and throughout the inside of the acini (Fig. 5, H and I, arrowheads). The localization of Laminin V revealed a multilayer epithelial morphology reminiscent of that described previously in *Drosophila* mutants [38]. The number of polarized versus unpolarized acini (based on Laminin V deposition) was quantified and, although greater than 90% of MC acini were polarized, less than 40% of either MH11 or MH2C were polarized (Table 1).

**Table 1:** Polarized localization of Laminin V is lost with Hugl knockdown.

Cell Line	LamV polarized	LamV unpolarized	n	% polarized	Fisher’s exact test
Control shRNA (MC)	51	3	54	94%	
Hugl1 shRNA (MH11)	23	38	61	38%	p<0.0001
Control shRNA (MC)	40	3	43	93%	
Hugl2 shRNA (MH2C)	11	26	37	30%	p<0.0001

Laminin V localization within acinar structures was scored as polarized (exclusively basal) or unpolarized (apical or luminal localization) and statistical significance was calculated using 2×2 contingency tables and Fisher’s Exact test. Data in table is reflective of one of three trials producing similar statistically significant results.

**Figure 6:**
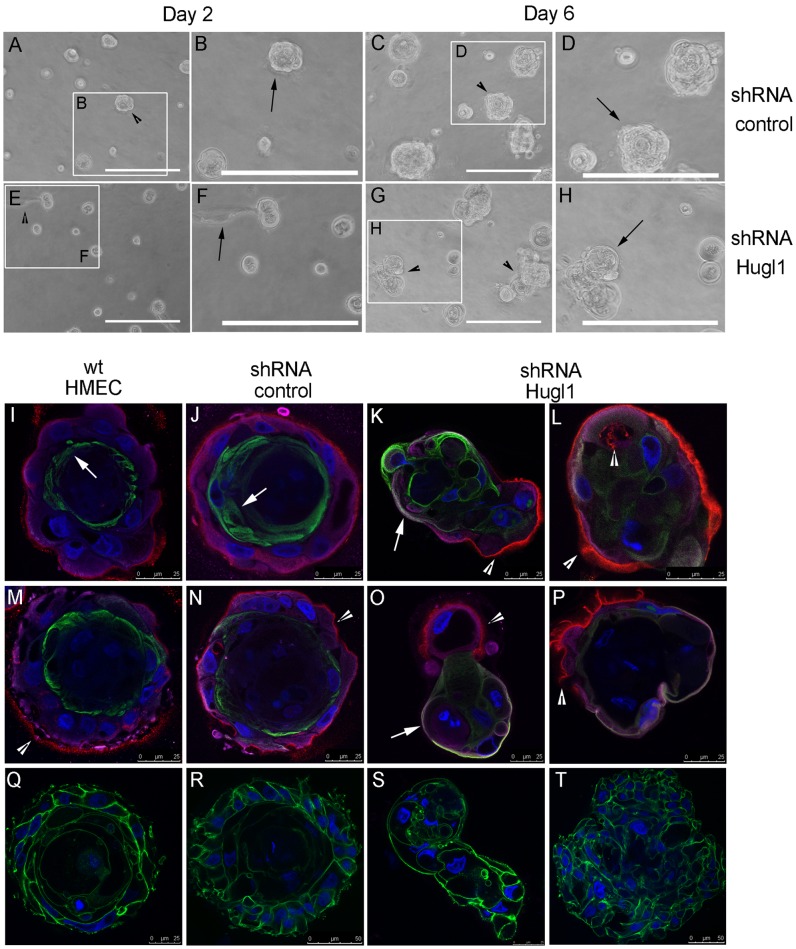
Hugl1 regulates membrane domain formation in HMECs. Control shRNA and Hugl1 shRNA HMECs were cultured in Matrigel for 6 days and imaged by bright field at 20X at day 2 and day 6 of growth. White boxes in A, C, E, and G outline areas magnified in B, D, F, and H. Wild type, control shRNA, and Hugl1 shRNA HMECs (I–T) were cultured in Matrigel for 6–10 days, fixed in 2% PFA, permeabilized, and incubated with primary antibodies to apical (anti-MUC1, green, I–P), basolateral (anti-Integrin α6, purple, I–P) and basal (anti-Laminin V, red, I–P) or cytoskeletal (anti-phalloidin, green, Q–T) domains followed by fluorescently labeled secondary antibodies (FITC, Alexa 488, 594, 647). Slides were mounted with antifade mounting media containing DAPI. Acini were imaged with a Leica confocal microscope to obtain equatorial cross-sectional images of their morphology at 630X. Arrows in I, J, K and O indicate MUC 1 localization. Arrowheads in K, L, M, N, O, and P indicate Laminin V localization. Scale bars indicate size in microns.

MCF10A cells, while able to form polarized acini in Matrigel, do not form tight junctions [39]. Therefore, to fully analyze alterations in apicobasal polarity, we also investigated the role of Hugl1 in Human Mammary Epithelial Cells (HMECs). HMECs were transduced with either control shRNA or Hugl1 shRNA-1 lentiviral particles, selected with puromycin, and knockdown was verified by immunoblot (Fig. 1, F). Within four days of transduction, cells were seeded in Matrigel and allowed to grow for 6 to 10 days. Immediately upon seeding (day 2), control cells formed similar size and number of colonies to knockdown cells (Fig. 6, A and E, arrowheads), although small filopodic projections were visible in knockdown acini but not controls (Fig. 6, B and F, arrows). By day 6, large colonies of cells were observed in both control and Hugl1 knockdown, although the Hugl1 knockdown colonies appeared less organized than the controls (Fig. 6, C and G, arrowheads). Specifically, most control acini had grown into discrete round colonies by this time, while knockdown colonies were less uniform, formed vacuoles, and formed clusters of smaller masses (Fig. 6, D and H, arrows).

To analyze apicobasal polarity in HMECs, localization of MUC1 (apical marker) Integrin α6 (basolateral marker), Laminin V deposition (basal marker) and DAPI nuclear stain was performed using four channel confocal microscopy. Cytoskeletal structure of F-actin was evaluated by phalloidin. In both wild type HMECs and shRNA control HMECs, apicobasal polarity was observed, with MUC1 (green) in the apical membrane (Fig. 6, I, J, M N, arrows), and Laminin V (red) and Integrin a6 (purple) at the basement membrane (Fig. 6, I, J M, N, arrowheads indicate Laminin V). Evaluation of the actin cytoskeleton in control acini highlighted an acinar structure with cells forming cell-cell adhesions around a central lumen (Fig. 6, R). Alternatively, knockdown of Hugl1 expression resulted in loss of a central lumen, as shown by actin localization (Fig. 6, S and T). Significantly, transmembrane proteins were no longer restricted to the apical or basolateral surfaces in the Hugl1 knockdown (Fig. 5, L, M, P, Q). With loss of Hugl1, MUC1 (green) and Integrin α6 (purple) were found colocalized (white) at multiple membrane surfaces (Fig. 6, K and O, arrows). Distinct Laminin V deposition was still observed relative to the cells (Fig. 6, K, L, O, P, arrowheads), and frequently appeared as fingerlike projections into the Matrigel (Fig. 6, P, arrowhead). In addition, clusters of cells, as opposed to forming acini, formed structures in which clumps of cells appeared to be migrating away from the Laminin V deposition (Fig. 6, K and O). Note that acini formation in HMECs was highly irregular and variable, therefore localization of polarity markers was not quantified. To demonstrate this variability, images K, L, O, and P from Figure 6 are replicate examples of structures formed in Hugl1 knockouts.

## Discussion

We have investigated the role of Hugl1 and Hugl2 in human breast epithelial cells and discovered that loss of either of these proteins from the mammary epithelium alters polarized 3-D acinar formation in Matrigel, resulting in a multi-layered tissue phenotype with random polarization of individual cells and loss of apical and basolateral membrane distinctions. These changes were associated with an increase in proliferation when epithelial cells are grown in a 3-dimensional extracellular matrix, but not when cells are grown on plastic. Alternatively, tumor cells grown on plastic in which Hugl1 expression has been exogenously restored, exhibit a decrease in proliferation. Loss of Hugl1 and Hugl2 is also associated with the induction of a phenotypic EMT. Overall, these results indicate that Hugl1 and Hugl2 promote a cellular program of polarized differentiation in mammary epithelium, and that their loss may allow for a proliferative mesenchymal fate to predominate.

While Hugl1 and Hugl2 knockdown in MCF10A cells resulted in similar phenotypes, all phenotypes were stronger in the Hugl1 knockdown. Hugl1 and Hugl2 also appear to have differential expression in breast epithelium. The difference in Hugl paralog expression between luminal cells and basal cells in the mammary epithelium indicates that they may have distinct roles in these two cell types, while maintaining similar abilities to drive polarity [29]. They also do not appear to be able to compensate for one another, as they do not both need to be knocked down to observe the phenotype. Hugl1 is expressed in both luminal and myoepithelial cells, while Hugl2 is predominantly expressed in myoepithelial cells, indicating a basal-cell specific function for Hugl2. In addition to loss of polarity and acinar overgrowth, we observed enlarged nuclei in the knockdown acinar structures. Alterations in nuclear size and shape remain a gold standard in cancer diagnosis, yet no solid mechanistic conclusions have been reached to explain the molecular basis of nuclear deformation in cancer cells [40].

The observation of altered Laminin V deposition in acini in the Hugl1 and Hugl2 knockdown cells is a phenotype that also can be induced by the constitutive activation of T-24 H-Ras resulting in the formation of tumors in immune-compromised mice [41], [42]. A possible mechanism for this Laminin V mislocalization is a disruption of vesicle trafficking to the basal membrane. It has been previously shown that overexpression of aPKCζ can interrupt polarization of mammary cells in 3-D culture and cause acinar overgrowths [43]. Similarities between Hugl1 and Hugl2 loss and oncogene overexpression indicate that Hugl driven polarity may be important in tumor suppression and future work will address this possibility. Overall, these studies demonstrate that Hugl1 and Hugl2 are key components of the polarity and differentiation programs in mammary gland epithelium.

## Supporting Information

Figure S1 3 µm normal human mammary tissue sections were incubated with anti-Hugl1 (A, Alexa 594 secondary, red), anti-Hugl2 (B, Alexa 594, red) and anti-cytokeratin 18 (A and B, Alexa 488, green). Single images were obtained at 400X on a Leica SP5 confocal microscope. Channels are separated to display differential expression of Hugl1 and Hugl2 in mammary tissue (C) Hugl1 (D) Hugl2 (E and F) cytokeratin 18 (luminal epithelial marker) (G and H) DAPI.
(TIF)

Table S1 Cleaved caspase 3 stained images of single acini were scored as either cleaved caspase positive or cleaved caspase negative. Categorical data on apoptotic activity were compared with 2×2 contingency tables and statistical significance was calculated with a Fisher’s exact test.
(DOCX)
