# Supplementary figures and images for: Hugl1 and Hugl2 in Mammary Epithelial Cells: Polarity, Proliferation, and Differentiation
Source: PLoS One. 2012 Oct 23;7(10):e47734. doi: 10.1371/journal.pone.0047734 (PMC3479147; doi:10.1371/journal.pone.0047734)

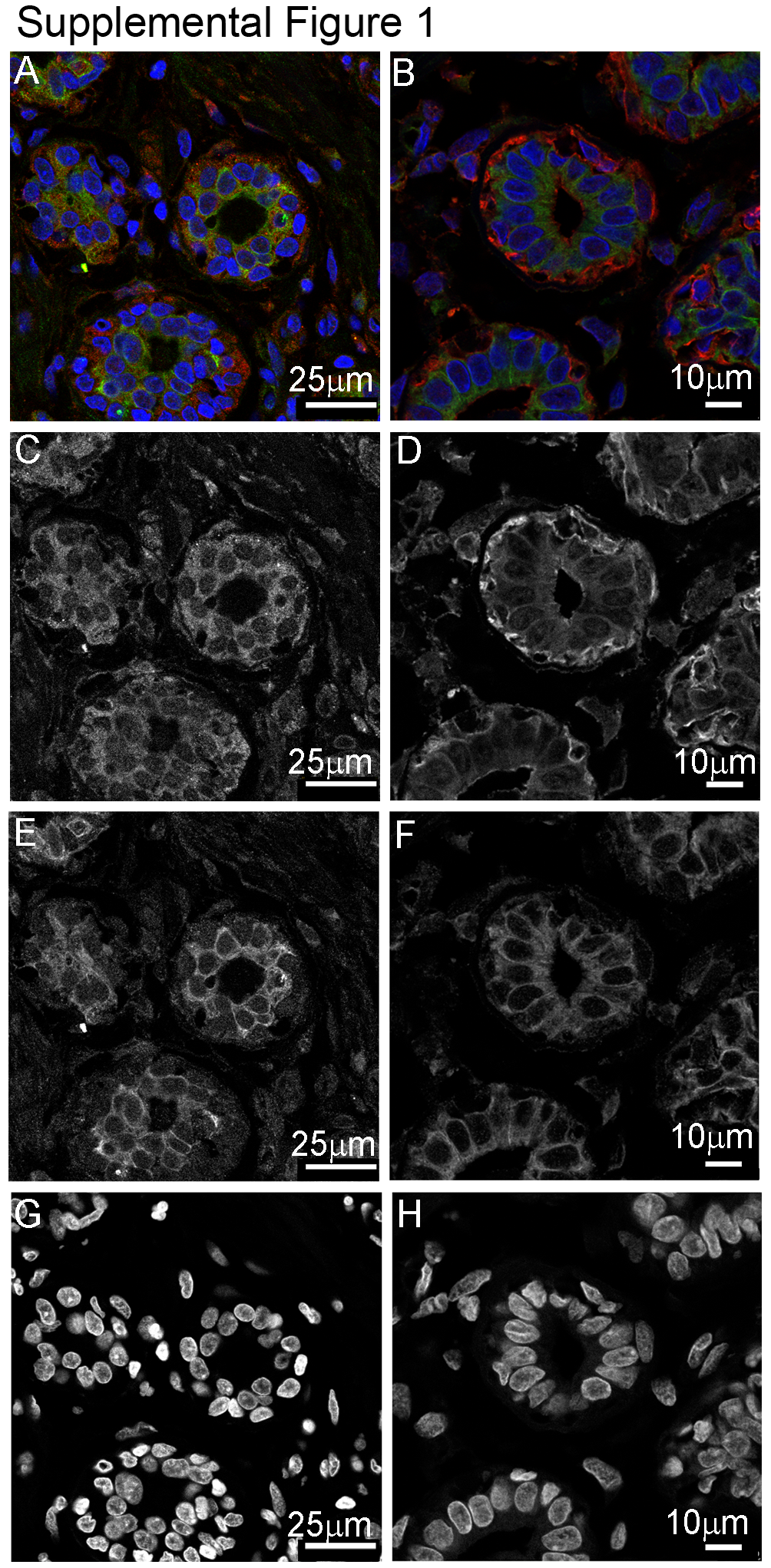

Supplement: Figure S1 — 3 µm normal human mammary tissue sections were incubated with anti-Hugl1 (A, Alexa 594 secondary, red), anti-Hugl2 (B, Alexa 594, red) and anti-cytokeratin 18 (A and B, Alexa 488, green). Single images were obtained at 400X on a Leica SP5 confocal microscope. Channels are separated to display differential expression of Hugl1 and Hugl2 in mammary tissue (C) Hugl1 (D) Hugl2 (E and F) cytokeratin 18 (luminal epithelial marker) (G and H) DAPI. (TIF) [file pone.0047734.s001.tif]
