# Supplementary material for: Hugl1 and Hugl2 in Mammary Epithelial Cells: Polarity, Proliferation, and Differentiation
Source: PLoS One. 2012 Oct 23;7(10):e47734. doi: 10.1371/journal.pone.0047734 (PMC3479147; doi:10.1371/journal.pone.0047734)
Supplement: Table S1 — Cleaved caspase 3 stained images of single acini were scored as either cleaved caspase positive or cleaved caspase negative. Categorical data on apoptotic activity were compared with 2×2 contingency tables and statistical significance was calculated with a Fisher’s exact test. (DOCX) [file pone.0047734.s002.docx]

**Supplemental Table 1.**

| Cell Line | CC 3 active | CC 3 inactive | n | % active caspase | Fisher’s exact test |
| --- | --- | --- | --- | --- | --- |
| Control shRNA (MC) | 56 | 10 | 66 | 85% |  |
| Hugl1 shRNA (MH11) | 27 | 10 | 37 | 73% | p = 0.1943 |
| Hugl2 shRNA (MH2C) | 27 | 8 | 35 | 77% | p = 0.4144 |
